# A High-Potential Phenoxazine Sulfonate Posolyte for Aqueous Zinc–Organic Flow Batteries

**DOI:** 10.3390/molecules31081337

**Published:** 2026-04-19

**Authors:** Guibao Wu, Linjing Miao, Mengna Qin, Qun Chen, Xiaofei Yu, Haiguang Gao, Juan Xu, Jianyu Cao

**Affiliations:** Jiangsu Key Laboratory of Advanced Catalytic Materials and Technology, School of Petrochemical Engineering, Changzhou University, Changzhou 213164, China

**Keywords:** aqueous zinc–organic flow batteries, phenoxazine sulfonate, posolyte, redox potential, aqueous solubility

## Abstract

Aqueous redox flow batteries (ARFBs) are a promising solution for large-scale energy storage; however, the development of organic posolytes that combine high redox potential with long-term stability remains a significant hurdle. This study introduces sodium 3-(10H-phenoxazin-10-yl)propane-1-sulfonate (POZS), a novel sulfonate-functionalized phenoxazine derivative designed to overcome these limitations. By incorporating hydrophilic anionic sulfonic groups, this molecular engineering strategy enhances the structural stability of redox-active phenoxazine materials. Although POZS shows limited solubility in pure water, its solubility increases to 0.98 M (equivalent to a charge capacity of 26.3 Ah L^−1^) upon the addition of 1.5 M tetraethylammonium chloride (TEAC). This enhancement suggests that the supporting electrolyte optimizes the ionic environment and mitigates intermolecular aggregation, thereby facilitating higher active species concentration. Electrochemical characterization of POZS reveals a highly positive redox potential of 1.51 V (vs. Zn/Zn^2+^) and rapid electron transfer kinetics (2.02 × 10^−2^ cm s^−1^). When tested in a zinc-based hybrid flow cell, the POZS posolyte demonstrates excellent rate capability (up to 50 mA cm^−2^) and a temporal capacity fade rate of 0.335% per hour over 500 cycles—a nearly five-fold improvement over previously reported quaternized phenoxazines. Post-cycling analyses indicate that while the phenoxazine core remains susceptible to nucleophilic ring substitution, the pendant sulfonate groups ensure that any resulting byproducts remain soluble, preventing the catastrophic depletion typically caused by the precipitation of degraded active species. These findings establish a robust molecular framework for the design of high-potential, durable organic posolytes for sustainable energy storage systems.

## 1. Introduction

Aqueous redox flow batteries (ARFBs) represent a highly effective and reliable class of large-scale energy storage devices. They have garnered significant attention due to their unique advantages, including the decoupling of power and energy, flexible modularity, high safety, excellent efficiency, and extended cycle life [1,2,3]. Although vanadium-based species are the most extensively studied electrolyte materials for ARFBs, several limitations impede their widespread adoption, which mainly include low energy density, the scarcity of vanadium, the corrosive nature of strong acidic media, and potential environmental impacts [4,5]. Consequently, research over the past decade has increasingly shifted toward organic electrolyte materials. These are considered promising alternatives because they are composed of earth-abundant elements, offer flexible molecular tunability, and possess potential biodegradability [6,7,8,9].

Recent research has yielded significant advancements in low-potential organic negolyte materials for ARFBs. Numerous viologen (Vi), anthraquinone (AQ), and phenazine (PZ) derivatives with meticulously designed molecular structures have demonstrated exceptional electrochemical performance and robust chemical stability [9,10,11,12,13,14,15,16]. However, developing high-potential organic posolyte materials without compromising their lifespan remains a major challenge, which is mainly due to the fact that high-redox-potential organic molecules are often unstable in aqueous solutions due to their electron-deficient nature, making them prone to side reactions such as disproportionation, hydrolysis, or electrophilic addition/substitution [8,17]. As a result, few redox-active molecules suitable for posolyte applications have been reported [18].

Phenoxazine (POZ)—a class of important heterocyclic compounds featuring a central oxazine ring fused with two aromatic rings—is one of compelling posolyte candidates for ARFBs. It is structurally similar to phenothiazine (PTZ), but replaces the sulfur atom at position 5 with oxygen [19]. Recent studies have explored POZ derivatives as ARFB electrolyte materials [18,20,21]. For instance, Li et al. reported Basic Blue 3 (BB3), a POZ-based organic dye capable of a reversible two-electron redox reaction in acidic ARFBs [20]. The high chemical/electrochemical stability of BB3 in acidic media allows this POZ-based ARFB cell to exhibit excellent cycling stability. However, BB3 has a relatively low redox potential of only 0.54 V (versus the standard hydrogen electrode, SHE), making it challenging to find suitable negolyte materials that can provide sufficient cell voltage, given the narrow electrochemical stability window of water.

Quaternization is one of the most effective molecular engineering strategies for developing POZ-based electrolyte materials, which can significantly enhance water solubility of POZ derivatives without compromising its electrochemical activity [21]. Our group previously synthesized a phenoxazine quaternary ammonium compound, NEt-POZ, for neutral-pH ARFBs [18]. While NEt-POZ demonstrated high water solubility (approximately 2.6 M in water), highly positive redox potential, and rapid kinetics, it suffered from rapid capacity decay during long-term charge–discharge cycling. This was mainly attributed to the disproportionation of radical di-cations (NEt-POZ^2+•^) formed by electrochemical oxidation of NEt-POZ, which produces unstable peroxidized tri-cations (NEt-POZ^3+^) that react with chloride ions to form insoluble polychlorinated derivatives.

To suppress these synergistic side reactions triggered by the disproportionation of the oxidized species of POZ compounds, in this work, we designed and synthesized a sulfonate-functionalized phenoxazine, sodium 3-(10H-phenoxazin-10-yl)propane-1-sulfonate (POZS). This was achieved via a facile one-step sulfonation approach, incorporating hydrophilic anionic sulfonic groups into the POZ nucleus. When utilized as a posolyte for aqueous zinc–organic flow batteries (AZFBs), POZS exhibits a highly positive redox potential (1.51 V versus Zn/Zn^2+^) and rapid redox kinetics (2.02 × 10^−2^ cm s^−1^). Furthermore, POZS displays significantly enhanced aqueous solubility in 1 M tetraethyl ammonium chloride (TEAC) solution, reaching 0.98 M—nearly four times its solubility in pure water (~0.26 M). A Zn//POZS AZFB full cell was assembled, with POZS as the posolyte, zinc chloride as the negolyte, and TEAC as the supporting electrolyte, and its battery performance was evaluated. The post-analyses results demonstrate that the introduction of anionic sulfonic groups effectively mitigates the degradation pathways observed in previous POZ derivatives.

## 2. Results

**Scheme 1:**
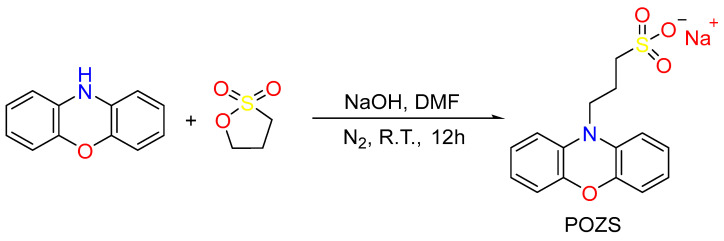
A synthetic route for POZS.

**Figure 1:**
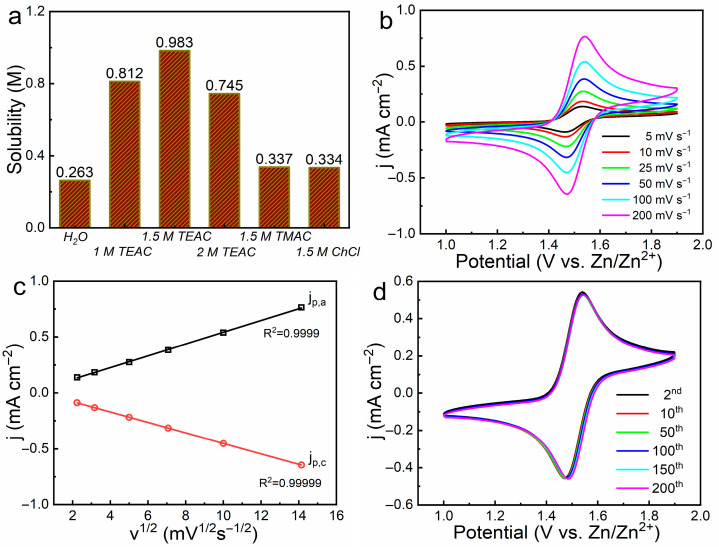
(**a**) Solubilities of the POZS in water and various quaternary ammonium salt solutions at room temperature. TEAC, tetraethylammonium chloride; TMAC, tetramethyl ammonium chloride; ChCl, choline chloride. (**b**) CVs of POZS (5 mM) in 1 M TEAC solution on a glassy carbon electrode at different scan rates. (**c**) The corresponding plots of peak current density (*j*_p_) versus square root of the scan rate (*v*^1/2^) for POZS. (**d**) CVs of POZS (5 mM) in 1 M TEAC solution on a glassy carbon electrode at a scan rate of 100 mV s^−1^ (up to 200 cycles).

POZS was synthesized by introducing water-soluble sulfonate groups into POZ molecules using 1,3-propanesultone as the sulfonate alkylating reactant (Scheme 1). Alkylation of POZ with 1.3 equivalents of 1,3-propanesultone in DMF for 12 h resulted in a POZS yield of 67.7%. ^1^H NMR analysis (Figure S1) confirmed the chemical structure of POZS. It is widely known that the parent POZ molecule has negligible solubility in water [22]. Although the introduction of hydrophilic sulfonic acid groups on the POZ cores significantly improved the water solubility of POZS, with a saturation concentration of 0.263 M (Figure 1a), this value is not sufficient for its ARFB application.

To enhance the solubility of POZS, three quaternary ammonium salts (i.e., TEAC, TMAC and ChCl) were developed as effective hydrophilic additives, which is not only due to the fact that their non-polar ethyl moieties can strongly interact with the phenoxazine units of POZS through short-range van der Waals forces based on the rule of the likes dissolve each other, but also because their highly ionized structure endows them with high water solubility and excellent ionic conductivity. It can be seen from Figure 1a and Figures S2–S6 that all three quaternary ammonium salts can increase the solubility of POZS. However, TEAC has a more significant effect in improving the solubility of POZS compared with TMAC and ChCl. This is especially the case when 1.5 M TEAC is added, and the solubility of POZS reaches 0.983 M, almost four times that in pure water, which corresponds to a theoretical charge capacity of 26.3 Ah L^−1^. Such a significant enhancement of POZS solubility by TEAC may be since the ethyl moieties of TEAC possess a stronger intermolecular interaction with POZ units compared to the methyl groups of TMAC or hydroxyethyl groups of ChCl. The pH of the POZS solution without supporting electrolytes is found to be 5.32 (Table S1). After adding 1 M TEAC, the pH slightly increases to 5.75. Further increasing the TEAC concentration hardly changes the pH. Notably, unlike TEAC, adding 1.5 M TMAC or 1.5 M ChCl to the POZS solution lowers the pH to around 4.60. This slight difference in pH may be related to the strength of the Coulombic interactions between POZS and different quaternary ammonium cations.

Figure 1b shows CVs of POZS on a glassy carbon electrode in 1 M TEAC solution at different scan rates. The standard redox potential (E^0^) for POZS is found to be about 1.51 V versus Zn/Zn^2+^. Therefore, for a Zn//POZS AZFB full cell, its POZS posolyte can theoretically deliver a specific energy of approximately 39.6 Wh kg^−1^ based on its cell voltage of 1.51 V and theoretical capacity of 26.3 Ah L^−1^. Notably, POZS exhibit a small peak separation (ΔE_p_ ~70 mV at 5 mV s^−1^) in 1 M TEAC solution, indicating their highly reversible redox kinetics. Moreover, the oxidation and reduction peak potentials hardly shift with an increasing scan rate. The peak currents of POZS give good linear relationships with the square root of the scan rate (ν^1/2^, Figure 1c), demonstrating that its redox kinetics in aqueous solution are controlled by diffusion. Using the Randles–Sevcik equation, the diffusion coefficients of POZS during the oxidation and reduction processes are calculated to be 1.64 × 10^−6^ and 1.10 × 10^−6^ cm^2^ s^−1^, respectively. Additionally, after repeating 200 CV cycles, it was observed that there was almost no decrease in the peak current of oxidation and reduction (Figure 1d), suggesting that POZS has excellent redox stability. Furthermore, based on the slope of the dependency between Ψ and ν^−1/2^, the kinetic rate constant (k^0^) of POZS estimated using Nicholson’s method is approximately 2.02 × 10^−2^ cm s^−1^ (Figure S7), again confirming its fast electron transfer kinetics. Moreover, parallel analysis of the blank solution reveals that the quaternary ammonium salt additive TEAC is non-redox active and does not interfere with the electrochemical property evaluation of POZS (Figure S8).

**Figure 2:**
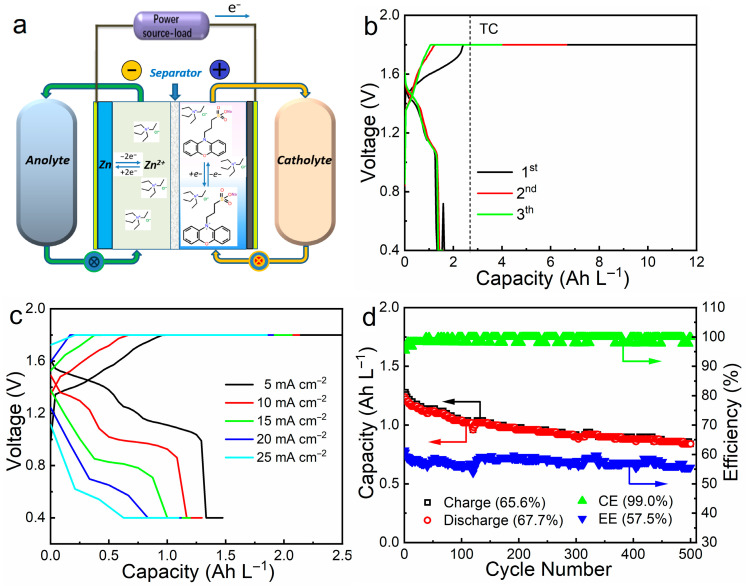
(**a**) Schematic representation of the Zn//POZS AHFB. (**b**) Voltage-capacity profiles of 0.1 M Zn//POZS AHFB cell at 5 mA cm^−2^ recorded at the 1st, 2nd and 3rd cycle, respectively. (**c**) Rate performance of this AHFB cell at different current densities. (**d**) Capacity and efficiency values of this AHFB cell at 10 mA cm^−2^ for 500 cycles. TC, theoretical capacity.

The attractive electrochemical/physicochemical properties of POZS, including relatively high solubility, suitable positive E^0^ (versus Zn/Zn^2+^) and rapid redox kinetics, prompt further evaluation of its energy storage performance in Zn-based ARFBs (Figure 2a). Therefore, a Zn//POZS ARFB full cell was assembled by employing a 0.1 M POZS solution containing 1.5 M TEAC and 0.2 M ZnCl_2_ (5 mL) as the posolyte and a mixed solution consisting only of 1.5 M TEAC and 0.2 M ZnCl_2_ (5 mL) as the negolyte, respectively. Notably, 1.5 M TEAC was added to the negolyte to maintain a general ion concentration balance on both sides of the separator, thereby preventing a concentration gradient-driven crossover of TEAC in the posolyte. The constant current and constant voltage (CCCV) protocol was adopted for the charge/discharge cycling experiments to accurately evaluate capacity fade and the ability to fully access available capacity.

Figure 2b shows voltage-capacity profiles of the 0.1 M Zn//POZS full cell for the initial three cycles at 5 mA cm^−2^. The charging capacities of the first three cycles are 12.0, 6.7, and 3.9 Ah L^−1^, significantly exceeding their theoretical capacity (TC, 2.68 Ah L^−1^). However, the discharge capacity stabilized at approximately 1.6 Ah L^−1^ (~60% of theoretical utilization). Figure 2c shows the voltage-capacity profiles for the 0.1 M Zn//POZS full cell at different current densities. The discharge capacity was ~1.48 Ah L^−1^ at 5 mA cm^−2^, slightly lower than the initial discharge capacity (~1.6 Ah L^−1^ at 5 mA cm^−2^) due to capacity degradation during the cycling process. As the current density increases from 5 to 25 mA cm^−2^, the discharge capacity decreases from 1.48 to 1.10 Ah L^−1^. Simultaneously, the average discharge voltage dropped from ~1.25 to ~0.45 V due to increased overpotential.

To assess the cycling stability and efficiencies of the POZS posolyte over repeated cycles, 500 charge/discharge cycles were performed for a 0.1 M Zn//POZS full cell at 10 mA cm^−2^ (Figure 2d and Figure S9). After an initial rapid decline in the first few dozen cycles, the decay rate in capacity stabilized. After the entire 500 cycles, the discharge capacity decreases from 1.24 to 0.84 Ah L^−1^, corresponding to a capacity retention of 67.7% (99.922% per cycle), or a temporal fade rate of ~0.335% per hour (or a total of 32.3% in 96.5 h). This temporal fade rate of the POZS posolyte is much lower than that of previously reported phenoxazine quaternary ammonium compound NEt-POZ at the same concentration (1.65% per hour [18]), demonstrating that the sulfonate group successfully inhibits rapid degradation during the electrochemical cycling process. The Coulombic efficiency (CE) and energy efficiency (EE) remain relatively stable throughout the entire 500 cycles, at 99.0% and 57.5%, respectively.

## 3. Discussion

**Figure 3:**
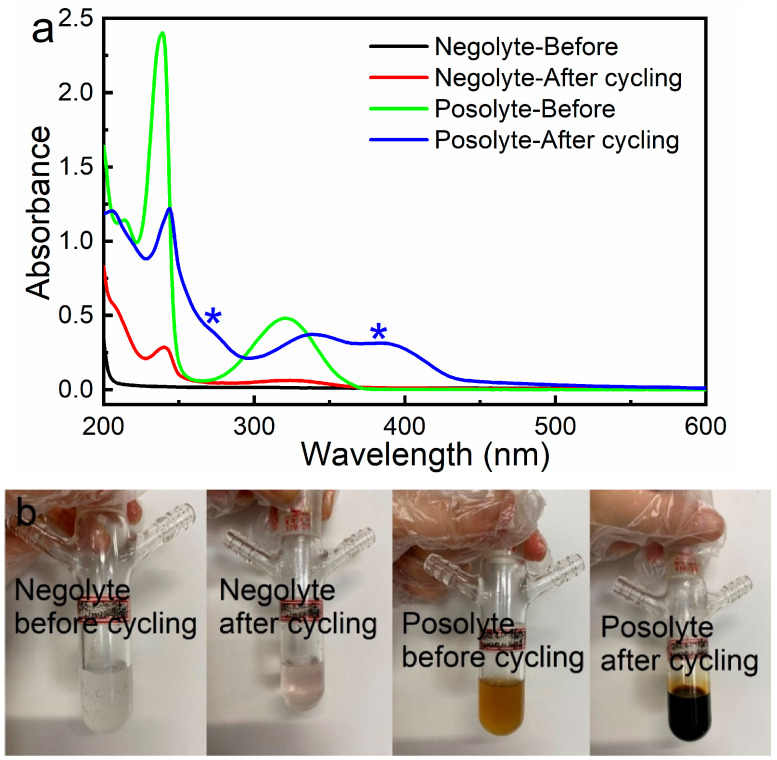
(**a**) UV-vis absorption spectra of both the posolyte and negolyte for 0.1 M Zn//POZS AHFB cell before and after cycling. The posolytes were diluted 1000-fold while the negolytes were diluted 100-fold for UV-vis measurements. (**b**) Images of the posolyte and negolyte before and after cycling.

To understand the capacity decay, UV-vis spectra of both the posolyte and negolyte were recorded before and after cycling (Figure 3a). After long-term cycling, the strong absorption peaks of POZS posolyte at 239 and 321 nm were red-shifted to 244 and 338 nm, respectively. Additionally, two new peaks at 270 and 383 nm (marked with blue asterisks in Figure 3a) appear for the cycled POZS posolyte. These UV-vis results indicate that POZS undergoes chemical side reactions during charge/discharge cycles, similar to the previously reported quaternized POZ molecules [18,21]. Furthermore, when comparing the UV-vis curve of the cycled negolyte with those of the posolyte and negolyte before cycling, it was found that there was ~11% POZS crossover from the posolyte to the negolyte side. A comparison of images of the posolyte and negolyte before and after cycling (Figure 3b) and the CV characterizations of the ZnCl_2_ negolyte before and after cycling (Figure S10) further verifies the presence of POZS crossover. Therefore, it can be concluded that the performance degradation is a combined result of chemical side reactions and crossover of POZS.

**Figure 4:**
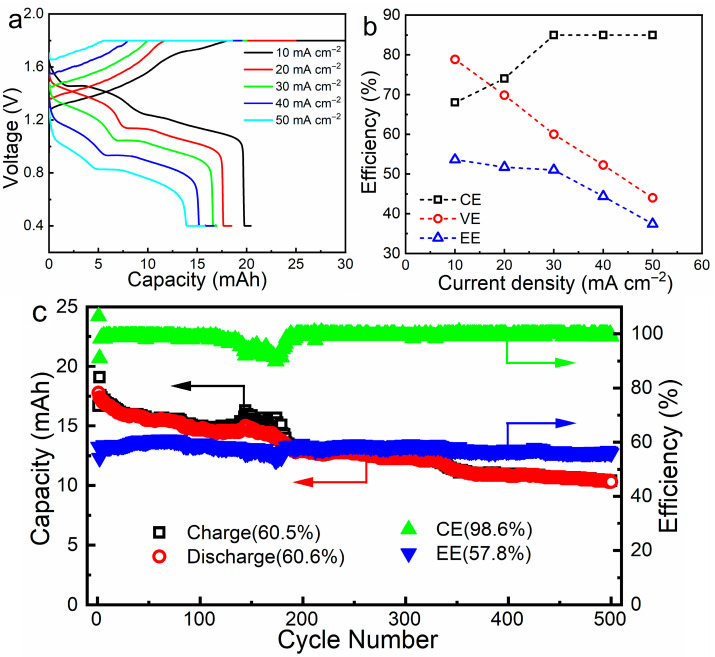
Battery performance of 0.5 M Zn//POZS AHFB cell. Capacities (**a**) and plots (**b**) of CE, VE, and EE at different current densities. (**c**) Cycling capacity and efficiency data of 0.5 M Zn//POZS AHFB cell at 30 mA cm^−2^ for 500 cycles.

To further evaluate the POZS posolyte, the battery performance at a higher concentration (0.5 M) was evaluated. As demonstrated in Figure 4a, a 0.5 M POZS posolyte exhibited a good rate capability. When the current density was increased from 10 to 20, 30, 40 and 50 mA cm^−2^, the discharge capacity decreased slightly from 20.4 to 18.5, 17.8, 16.7 and 15.8 mAh. This represents a 77.5% retention of its initial capacity, confirming the fast charge-transfer kinetics of POZS even at elevated concentrations. As the current density increases, the efficiency metrics change in a trend that meets expectations (Figure 4b). The CE increases with the rise in current density, likely due to a reduction in crossover time, while the VE and EE values decrease because of increased ohmic polarization. Notably, even at higher current densities, the CE for the 0.5 M Zn//POZS AHFB cell is only ~85%, indicating that side reactions continuously occur during the rate capability experiments.

The cycling performance for the 0.5 M Zn//POZS full cell was subsequently evaluated at 30 mA cm^−2^ (Figure 4c). This 0.5 M cell shows stable cycling in a period of 86.7 h for 500 cycles. During long-term cycling, 60.6% of the initial discharge capacity is retained, corresponding to a capacity retention rate of 99.900% per cycle (or a temporal fade rate of about 0.454% per hour), indicating that the 0.5 M POZS posolyte exhibits good cycling stability. Interestingly, the average CE consistently remains at 98.6% during long-term cycling, significantly higher than the 85% observed in the initial rate performance tests, suggesting that side reactions are greatly reduced in the 0.5 M Zn//POZS full cell during subsequent long-term cycling, and the system tends to stabilize as the cycling progresses. Over 500 cycles, the average EE is about 57.8%, which is also significantly higher than the approximately 51% obtained in the rate performance tests. Post-cycling observations revealed that some dark purple oily precipitates appeared in the cycled POZS posolyte, partially adhering to the walls of the storage tank and pipes (Figure S11a). After adding 5 mL of water, these precipitates completely dissolved (Figure S11b), indicating that the precipitates from the cycled POZS posolyte are still water-soluble, albeit with relatively low solubility.

**Figure 5:**
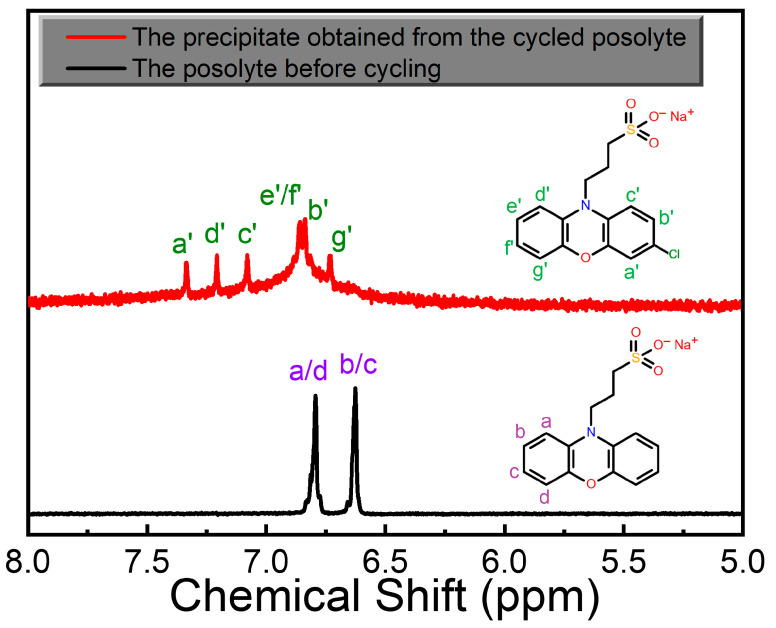
^1^H NMR spectra of the POZS posolyte before cycling and the product that precipitates from the POZS posolyte after cycling.

To identify possible side-reaction products occurring in the charge–discharge cycling of POZS, ^1^H NMR analyses were carried out. Figure 5 shows ^1^H NMR spectrum of the product that precipitates from the cycled POZS posolyte. Significantly different from the POZS posolyte before cycling, six distinct aromatic proton peaks were observed in the ^1^H NMR spectrum of the precipitate product, which are located at chemical shifts of 6.74, 6.84, 6.86, 7.08, 7.21, and 7.34 ppm, respectively. All of these ^1^H NMR signal peaks can be attributed to the aromatic protons of mono-chlorinated POZS, which indicates that POZS species undergo nucleophilic ring-substitution reactions during operation. This substitution mechanism is further supported by a significant decrease in posolyte pH, which dropped from 5.96 to 3.08 due to the accumulation of protons as byproducts of nucleophilic ring-substitution reaction [18,21]. Notably, despite these side effects, POZS demonstrates superior stability compared to its predecessors. This is attributed to the fact that the chlorinated derivatives of POZS remain more soluble in aqueous electrolytes, preventing the catastrophic depletion of active species seen in other phenoxazine systems.

## 4. Materials and Methods

### 4.1. Materials

Phenoxazine (POZ, 98%, Aladdin, Riverside, CA, USA), 1,3-propanesultone (99%, Aladdin), sodium hydroxide (NaOH, Aladdin), dichloromethane (Aladdin), N,N-dimethylformamide (DMF, anhydrous, Aldrich, St. Louis, MO, USA), tetraethyl ammonium chloride (TEAC, Aldrich), tetramethyl ammonium chloride (TMAC, Aldrich) and choline chloride (ChCl, Aldrich) were used as received.

### 4.2. Synthesis of POZS

To a 50 mL round-bottomed flask, we added POZ (1.0 g, 5.46 mmol), NaOH (0.28 g, 7.10 mmol) and dry DMF (20 mL). The mixture was stirred under a nitrogen atmosphere for one hour at room temperature. 1,3-Propanesultone (0.87 g, 7.10 mmol) was then added slowly, and the reaction continued under a nitrogen atmosphere for 12 h at room temperature. After completion, 100 mL of dichloromethane followed by 100 mL of water were added sequentially. The mixture was allowed to stand for 3 h after intense stirring. The organic phase was separated, and the aqueous phase was washed with an additional 50 mL of dichloromethane. The combined organic extracts were concentrated by rotary evaporation to yield a crude product. This crude product was dissolved in 50 mL of ethyl acetate, and the orange–yellow solid product (1.21 g, 67.7% yield) was isolated via suction filtration and dried under a vacuum at 60 °C.

### 4.3. Characterization

The ^1^H nuclear magnetic resonance (NMR) spectra were determined on a Bruker ARX-500 type NMR spectrometer (Bruker, Billerica, MA, USA). The solubility experiments of POZS were carried out on a Shimadzu UV mini 1240 UV-vis spectrophotometer (Shimadzu, Tokyo, Japan) at room temperature. The linear relation between absorbance and concentration was established at a peak wavelength of 321 nm.

### 4.4. Electrochemical Measurements

Cyclic voltammetry (CV) experiments were conducted on a CHI 660B electrochemical working station (CH Instruments, Austin, TX, USA) using a conventional three-electrode system under a nitrogen atmosphere at room temperature, where a polished glassy carbon (GC, 0.0707 cm^2^) electrode served as the working electrode while a zinc electrode serves as both the counter electrode and reference electrode, respectively. Before measurements, the electrolyte solution underwent a nitrogen gas purge for about 20 min to eliminate dissolved oxygen.

### 4.5. Hybrid-Flow Cell Tests

A hybrid-flow cell was assembled using commercially available flow cell hardware (5 cm^2^, Fuel Cell Technologies Inc., Albuquerque, NM, USA), which includes metallic end plates, gold-plated current collectors, and graphite flow field plates with serpentine flow channels. All the flow cell measurements were performed on a Neware battery testing system. A piece of Nafion 212 cation exchange membrane was utilized as the separator to separate the negolyte and posolyte. Graphite felts (2 mm thick, CeTech, Taichung, Taiwan) were utilized as electrodes. For the 0.1 M cell, a 0.1 M POZS in 1.5 M TEAC + 0.2 M ZnCl_2_ solution (5 mL) was used as the posolyte while a 1.5 M TEAC + 0.2 M ZnCl_2_ solution (5 mL) was used as the negolyte. For the 0.5 M cell, a 0.5 M POZS in 1.5 M TEAC + 0.5 M ZnCl_2_ solution (5 mL) was used as the posolyte while a 1.5 M TEAC + 0.5 M ZnCl_2_ solution (5 mL) was used as the negolyte. Both the posolyte and negolyte were kept under a nitrogen atmosphere, and the flow rate was maintained at 60 mL min^−1^ using peristaltic pumps. The charge–discharge cycling tests were carried out at different current densities by employing the constant current followed by constant voltage (CCCV) protocol, with voltage cut-offs of 0.4 and 1.8 V.

## 5. Conclusions

In summary, we have successfully designed and synthesized a novel sulfonate-functionalized phenoxazine derivative, POZS, as a high-potential posolyte for aqueous zinc–organic flow batteries. The introduction of anionic sulfonic acid groups onto the phenoxazine core significantly enhances its electrochemical stability compared to previously reported quaternized derivatives. By utilizing TEAC as a supporting electrolyte, we achieved a high solubility of 0.98 M, leveraging intermolecular van der Waals interactions to overcome the inherent limitations of the POZ structure in aqueous media. The POZS posolyte demonstrated a highly positive redox potential of 1.51 V vs. Zn/Zn^2+^, rapid electron transfer kinetics (2.02 × 10^−2^ cm s^−1^), and impressive rate capability of up to 50 mA cm^−2^. Most importantly, long-term cycling tests revealed a temporal capacity fade rate of only 0.335% h^−1^—a nearly five-fold improvement over the non-sulfonated NEt-POZ benchmark. Our post-cycling analysis via 1H NMR and UV-vis spectroscopy confirmed that while nucleophilic ring substitution remains the primary degradation pathway, the sulfonate groups render the resulting byproducts more water-soluble. This alleviates the precipitation phenomenon that typically plague phenoxazine-based ARFBs. This work provides a clear molecular engineering strategy for developing stable, high-potential organic electrolytes, moving a step closer to the practical deployment of sustainable, large-scale aqueous energy storage systems.

## Data Availability

The original contributions presented in this study are included in the article and Supplementary Materials. Further inquiries can be directed to the corresponding authors.

## Supplementary Materials

The following supporting information can be downloaded at https://www.mdpi.com/article/10.3390/molecules31081337/s1, Figure S1: ^1^H NMR spectrum of POZS (500 MHz, D_2_O); Figure S2: (a) UV-vis absorption spectra of the POZS aqueous solutions (added 1 M TEAC) with different concentrations. (b) The linear plot between the concentration and the maximum absorbance recorded at a wavelength of 321 nm; Figure S3: (a) UV-vis absorption spectra of the POZS aqueous solutions (added 1.5 M TEAC) with different concentrations. (b) The linear plot between the concentration and the maximum absorbance recorded at a wavelength of 321 nm; Figure S4: (a) UV-vis absorption spectra of the POZS aqueous solutions (added 2 M TEAC) with different concentrations. (b) The linear plot between the concentration and the maximum absorbance recorded at a wavelength of 320 nm; Figure S5: (a) UV-vis absorption spectra of the POZS aqueous solutions (added 1.5 M TMAC) with different concentrations. (b) The linear plot between the concentration and the maximum absorbance recorded at a wavelength of 321 nm; Figure S6: (a) UV-vis absorption spectra of the POZS aqueous solutions (added 1.5 M ChCl) with different concentrations. (b) The linear plot between the concentration and the maximum absorbance recorded at a wavelength of 321 nm; Table S1: The pHs of POZS solutions with and without various quaternary ammonium supporting salt electrolytes; Figure S7: (a,b) CVs of POZS (5 mM) in 1 M TEAC solution at different scan rates (the scan rates (ν) from 5 to 5000 mV s^−1^). (c) Plot of Ψ versus ΔE. (d) Plot of Ψ versus ν^−1/2^; Figure S8: CVs of 1 M TEAC solutions with and without POZS (5 mM) at a scan rate of 25 mV s^−1^; Figure S9: The voltage-capacity profiles of 0.1 M Zn//POZS AHFB cell at 10 mA cm^−2^ during 500 charge-discharge cycles; Figure S10: CVs of the 0.2 M ZnCl_2_ negolyte before and after cycling and the 0.1 M POZS posolyte before cycling obtained at 25 mVs^−1^ on the GC electrode. The posolyte was diluted 20-fold for the CV measurement; Figure S11: (a) Photograph of the 0.5 M POZS posolyte after cycling, showing the dark purple, oily solid residue. (b) Photograph of this posolyte after adding 5 mL of water.

## References

[1] Li Z., Lu Y.-C. (2020). Material design of aqueous redox flow batteries: Fundamental challenges and mitigation strategies. Adv. Mater..

[2] Zhu F., Guo W., Fu Y. (2023). Functional materials for aqueous redox flow batteries: Merits and applications. Chem. Soc. Rev..

[3] Zhang J., Jiang G., Xu P., Kashkooli A.G., Mousavi M., Yu A., Chen Z. (2018). An all-aqueous redox flow battery with unprecedented energy density. Energy Environ. Sci..

[4] Rodby K.E., Jaffe R.L., Olivetti E.A., Brushett F.R. (2023). Materials availability and supply chain considerations for vanadium in grid-scale redox flow batteries. J. Power Sources.

[5] Cui Y., Zheng K., Yuan Z., Guo D., Xu J., Yu X., Zang J., Cao J. (2025). A multi-substituted phenazine derivative aqueous redox flow battery with high energy efficiency and long lifetime. J. Power Sources.

[6] Winsberg J., Hagemann T., Janoschka T., Hager M.D., Schubert U.S. (2016). Redox-flow batteries: From metals to organic redox active materials. Angew. Chem. Int. Ed..

[7] Cao J., Tian J., Xu J., Wang Y. (2020). Organic flow batteries: Recent progress and perspectives. Energy Fuel.

[8] Liu Y., Chen Q., Sun P., Li Y., Yang Z., Xu T. (2021). Organic electrolytes for aqueous organic flow batteries. Mater. Today Energy.

[9] Luo J., Hu B., Debruler C., Liu T.L. (2018). A π-conjugation extended viologen as a two-electron storage anolyte for total organic aqueous redox flow batteries. Angew. Chem. Int. Ed..

[10] Tang G., Peng K., Liu Y., Fang J., Wu W., Yang Z., Xu T. (2025). Propylene-bridged associative bis(bipyridinium) electrolytes for long-lifetime aqueous organic redox flow batteries. Angew. Chem. Int. Ed..

[11] Liu T., Wei X., Nie Z., Sprenkle V., Wang W. (2016). A total organic aqueous redox flow battery employing a low cost and sustainable methyl viologen anolyte and 4-HO-TEMPO catholyte. Adv. Energy Mater..

[12] Huskinson B., Marshak M.P., Suh C., Er S., Gerhardt M.R., Galvin C.J., Chen X., Aspuru-Guzik A., Gordon R.G., Aziz M.J. (2014). A metal-free organic-inorganic aqueous flow battery. Nature.

[13] Liu Y., Zhang P., Wu Z., Ding G., Song X., Ma J., Wang W., Wang X.-Z., Jin Z. (2024). Biomimetic naphthoquinone zwitterion derivative with water-solubilizing amino acid side chain for high-stability aqueous redox flow batteries. ACS Energy Lett..

[14] Liu Y., Zhang P., Wu Z., Wei J., Ding G., Song X., Ma J., Wang W., Jin Z. (2024). Screening ultra-stable (phenazine)dioxyalkanocic acids with varied water-solubilizing chain lengths for high-capacity aqueous redox flow batteries. J. Am. Chem. Soc..

[15] Hollas A., Wei X., Murugesan V., Nie Z., Li B., Reed D., Liu J., Sprenkle V., Wang W. (2018). A biomimetic high-capacity phenazine-based anolyte for aqueous organic redox flow batteries. Nat. Energy.

[16] Li L., Yao E., Ji Y., Wang P. (2025). A resonance hybrid design for stable aqueous organic redox flow batteries. Angew. Chem. Int. Ed..

[17] Tong L., Jing Y., Gordon R.G., Aziz M.J. (2019). Symmetric all-quinone aqueous battery. ACS Appl. Energy Mater..

[18] Qin M., Wu G., Zheng K., Yu X., Xu J., Cao J. (2024). A highly water-soluble phenoxazine quaternary ammonium compound catholyte for pH-neutral aqueous organic redox flow batteries. J. Energy Storage.

[19] Buene A.F., Almenningen D.M. (2021). Phenothiazine and phenoxazine sensitizers for dye-sensitized solar cells—An investigative review of two complete dye classes. J. Mater. Chem. C.

[20] Li H., Fan H., Ravivarma M., Hu B., Feng Y., Song J. (2020). A stable organic dye catholyte for long-life aqueous flow batteries. Chem. Commun..

[21] Fang X., Zeng L., Li Z., Robertson L.A., Shkrob I.A., Zhang L., Wei X. (2023). A cooperative degradation pathway for organic phenoxazine catholytes in aqueous redox flow batteries. Next Energy.

[22] Martínez-González E., Amador-Bedolla C., Ugalde-Saldivar V.M. (2022). Reversible redox chemistry in a phenoxazine-based organic compound: A two-electron storage negolyte for alkaline flow batteries. ACS Appl. Energy Mater..

