# Supplementary material for: A High-Potential Phenoxazine Sulfonate Posolyte for Aqueous Zinc–Organic Flow Batteries
Source: Molecules. 2026 Apr 19;31(8):1337. doi: 10.3390/molecules31081337 (PMC13118284; doi:10.3390/molecules31081337)
Supplement: Supplementary file 1 [file molecules-31-01337-s001.zip › molecules-4253140-supplementary.pdf]

Supporting Information

A High-Potential Phenoxazine Sulfonate Posolyte for Aqueous Zinc–Organic Flow Batteries

Guibao Wu[†], Linjing Miao[†], Mengna Qin[†], Qun Chen*, Xiaofei Yu, Haiguang Gao, Juan Xu and Jianyu Cao*

Jiangsu Key Laboratory of Advanced Catalytic Materials and Technology, School of Petrochemical Engineering, Changzhou University, Changzhou 213164, China

[†]These authors contributed equally to this work.

[*]Corresponding author,

E-mail: jycao@cczu.edu.cn; chenqun@cczu.edu.cn

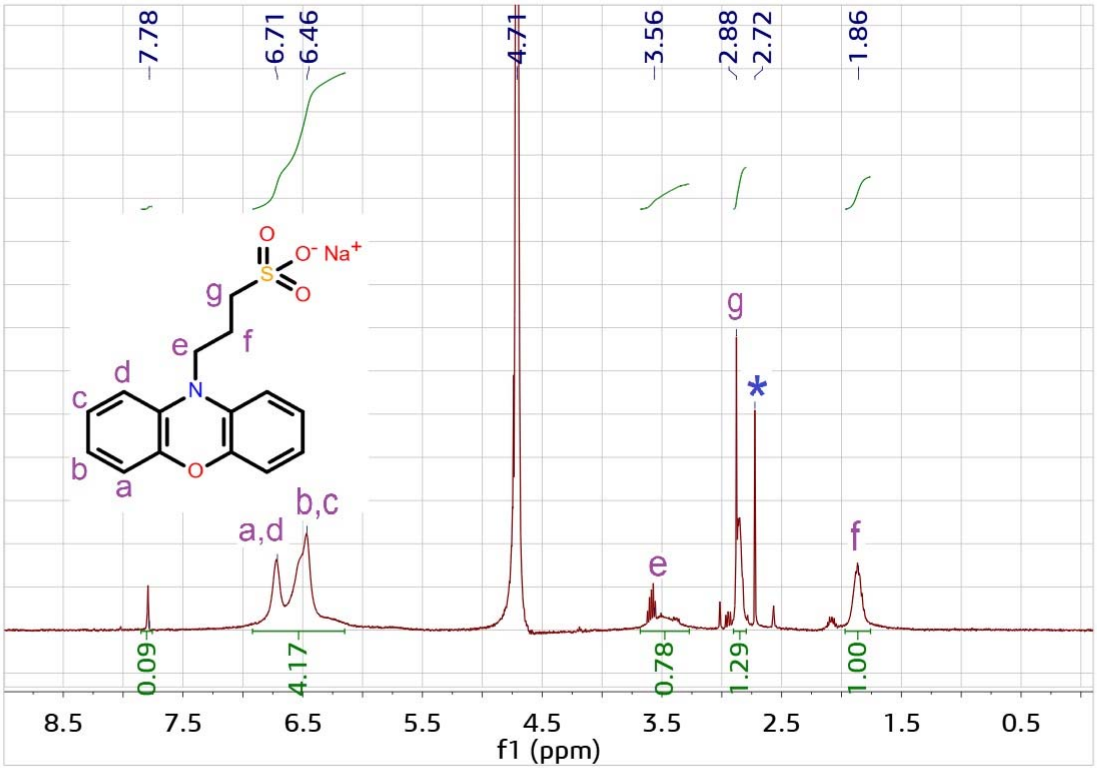

Fig. S1 ^{1}H NMR spectrum of POZS (500 MHz, D_2O). The proton peak marked with an asterisk comes from the residual solvent DMF.

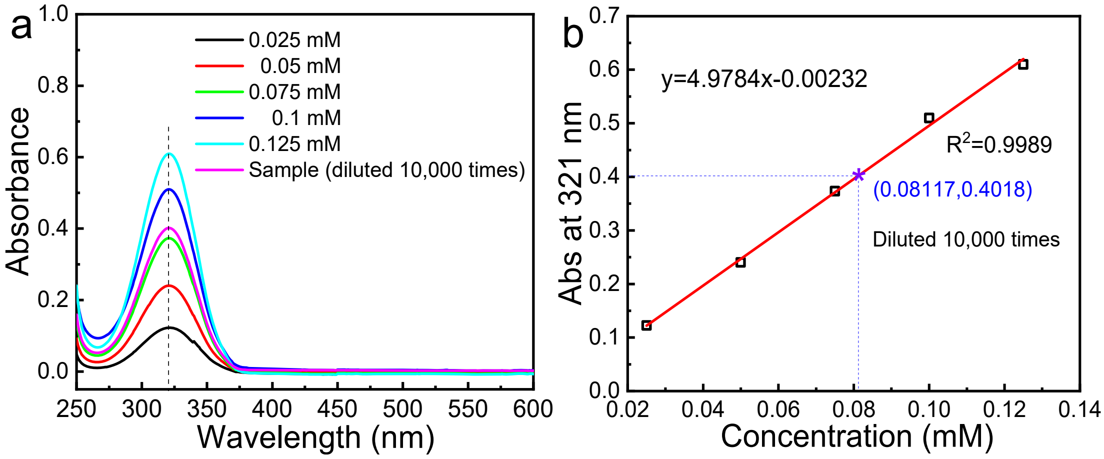

Fig. S2 (a) UV-vis absorption spectra of the POZS aqueous solutions (added 1 M TEAC) with different concentrations. (b) The linear plot between the concentration and the maximum absorbance recorded at a wavelength of 321 nm.

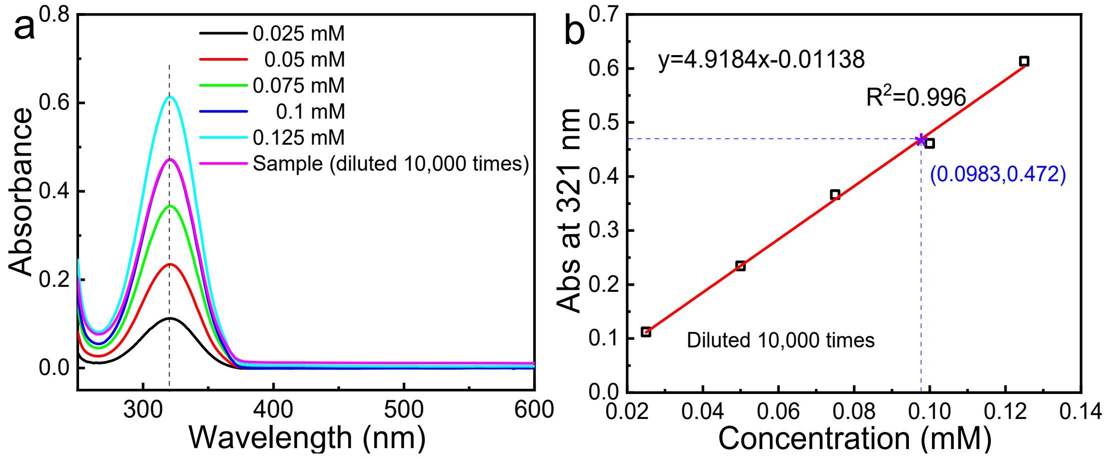

Fig. S3 (a) UV-vis absorption spectra of the POZS aqueous solutions (added 1.5 M TEAC) with different concentrations. (b) The linear plot between the concentration and the maximum absorbance recorded at a wavelength of 321 nm.

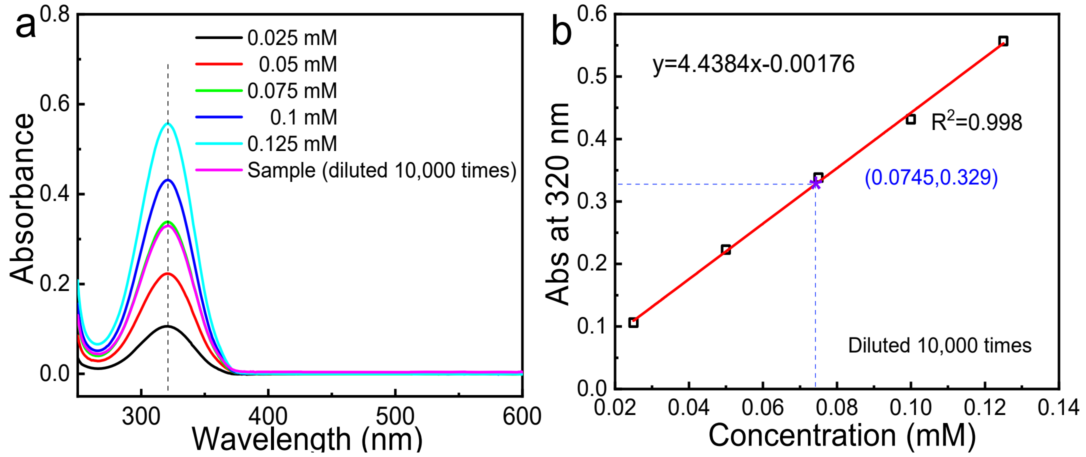

Fig. S4 (a) UV-vis absorption spectra of the POZS aqueous solutions (added 2 M TEAC) with different concentrations. (b) The linear plot between the concentration and the maximum absorbance recorded at a wavelength of 320 nm.

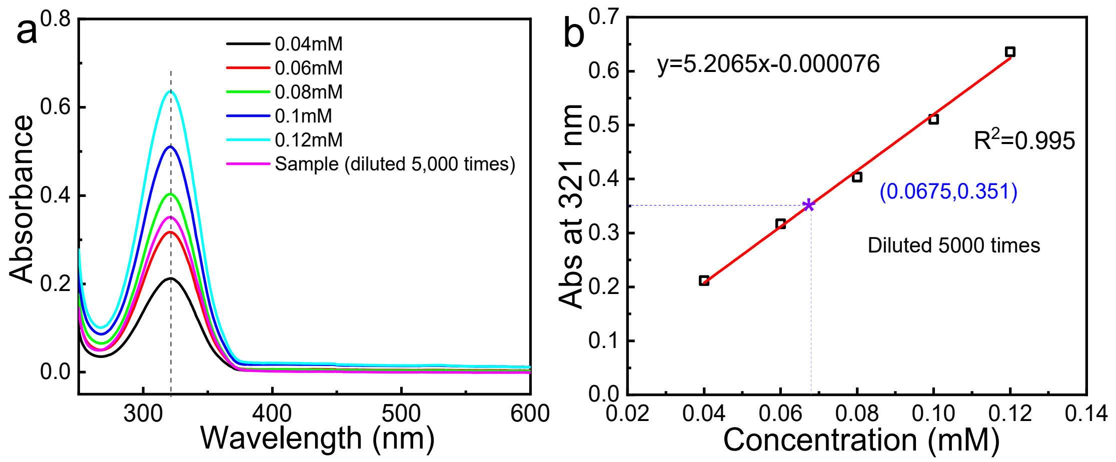

Fig. S5 (a) UV-vis absorption spectra of the POZS aqueous solutions (added 1.5 M TMAC) with different concentrations. (b) The linear plot between the concentration and the maximum absorbance recorded at a wavelength of 321 nm.

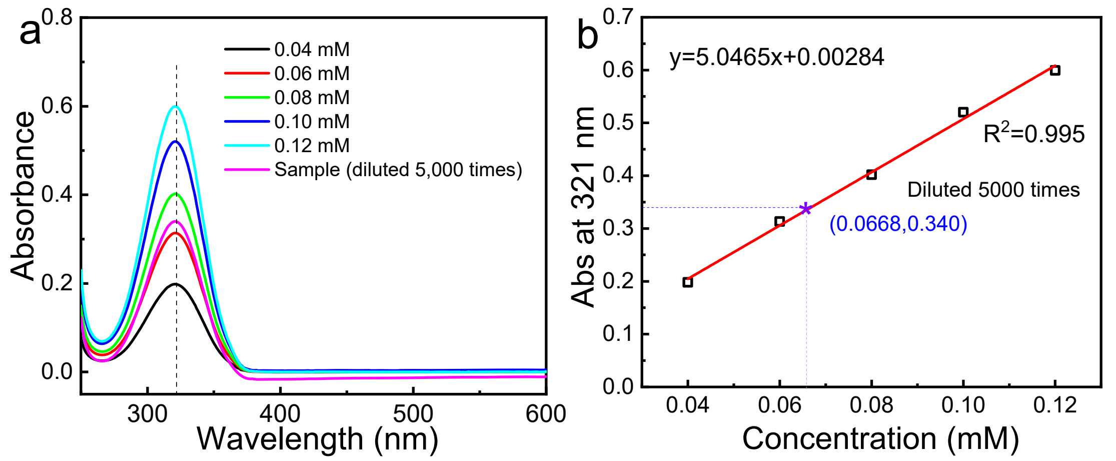

Fig. S6 (a) UV-vis absorption spectra of the POZS aqueous solutions (added 1.5 M ChCl) with different concentrations. (b) The linear plot between the concentration and the maximum absorbance recorded at a wavelength of 321 nm.

Table S1 The pHs of POZS solutions with and without various quaternary ammonium supporting salt electrolytes.

Sample	POZS in water	POZS in 1 M TEAC	POZS in 1.5 M TEAC	POZS in 2 M TEAC	POZS in 1.5 M TMAC	POZS in 1.5 M ChCl
pH	5.32	5.75	5.72	5.65	4.60	4.61

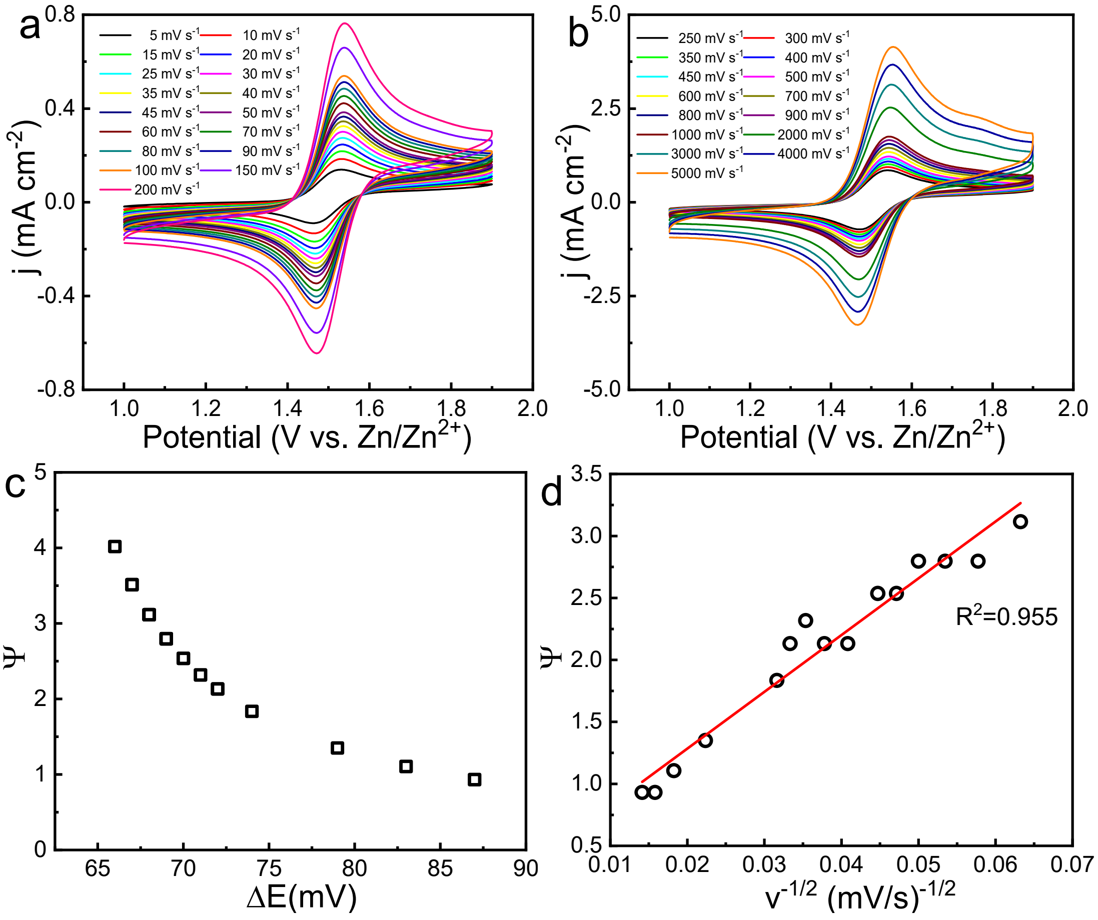

Fig. S7 (a-b) CVs of POZS (5 mM) in 1 M TEAC solution at different scan rates (the scan rates (ν) from 5 to 5000 mV s^{-1}). (c) Plot of *Ψ* versus Δ*E*. (d) Plot of *Ψ* versus $\nu^{-1/2}$.

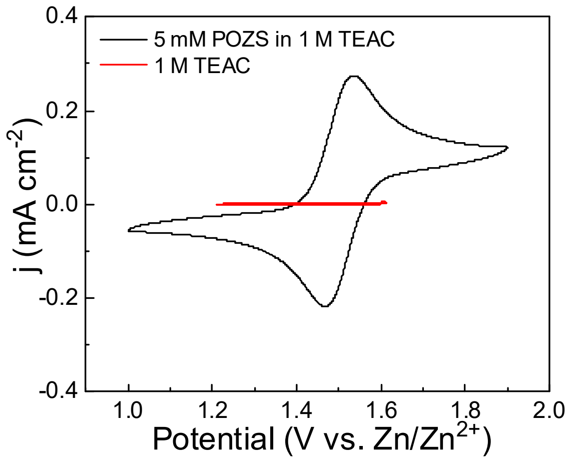

Fig. S8 CVs of 1 M TEAC solutions with and without POZS (5 mM) at a scan rate of 25 mV s^{-1}.

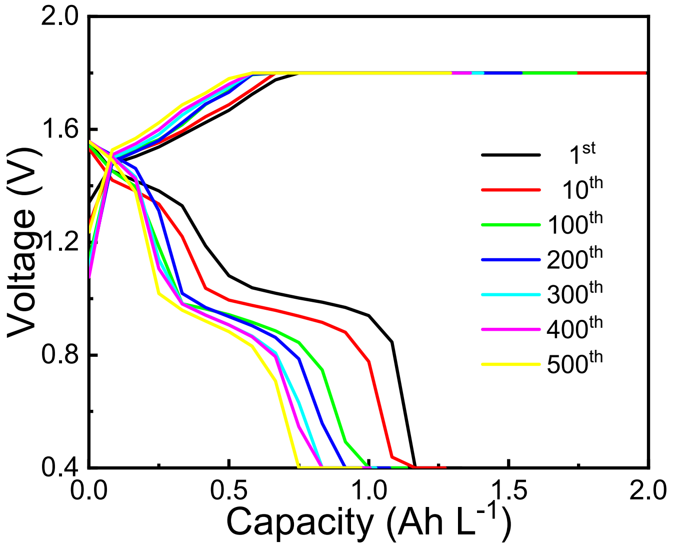

Fig. S9 The voltage-capacity profiles of 0.1 M Zn//POZS AHFB cell at 10 mA cm^{-2} during 500 charge-discharge cycles.

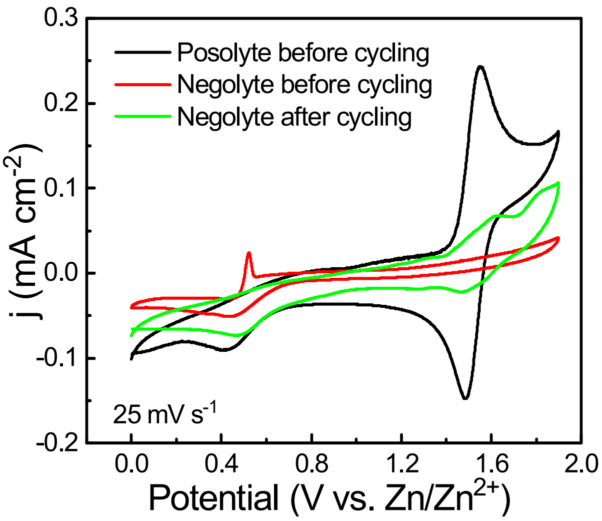

Fig. S10 CVs of the 0.2 M $ZnCl_2$ negolyte before and after cycling and the 0.1 M POZS posolyte before cycling obtained at 25 mVs^{-1} on the GC electrode. The posolyte was diluted 20-fold for the CV measurement.

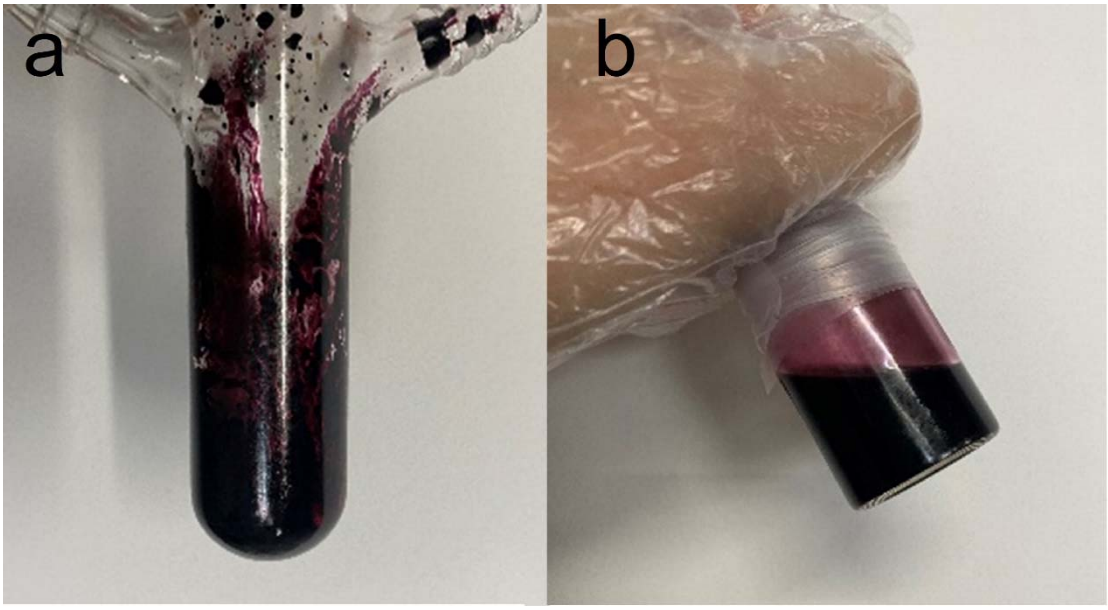

0.5 M posolyte after cycling

0.5 M posolyte after cycling (adding water)

Fig. S11 (a) Photograph of the 0.5 M POZS posolyte after cycling, showing the dark purple, oily solid residue. (b) Photograph of this posolyte after adding 5 ml of water. Our studies suggest that the residue is water-soluble, but the solubility is lower.
